# Metal nanoparticles: understanding the mechanisms behind antibacterial activity

**DOI:** 10.1186/s12951-017-0308-z

**Published:** 2017-10-03

**Authors:** Yael N. Slavin, Jason Asnis, Urs O. Häfeli, Horacio Bach

**Affiliations:** 1 Department of Medicine, Division of Infectious Diseases, University of British Columbia, 410-2660 Oak St., Vancouver, BC V6H3Z6 Canada; 2 Faculty of Pharmaceutical Sciences, University of British Columbia, Vancouver, BC Canada

**Keywords:** Nanoparticles, Metals, ROS, Mechanism of defense, Bacteria, Transcriptomics, Proteomics, Gene regulation, Antibacterial resistance

## Abstract

As the field of nanomedicine emerges, there is a lag in research surrounding the topic of nanoparticle (NP) toxicity, particularly concerned with mechanisms of action. The continuous emergence of bacterial resistance has challenged the research community to develop novel antibiotic agents. Metal NPs are among the most promising of these because show strong antibacterial activity. This review summarizes and discusses proposed mechanisms of antibacterial action of different metal NPs. These mechanisms of bacterial killing include the production of reactive oxygen species, cation release, biomolecule damages, ATP depletion, and membrane interaction. Finally, a comprehensive analysis of the effects of NPs on the regulation of genes and proteins (transcriptomic and proteomic) profiles is discussed.

## Background

As the field of nanomedicine emerges, there is a deficiency of research surrounding the topic of nanoparticle (NP) toxicity, particularly concerned with mechanisms of action. NPs have increasingly been used in industry over the past few decades with usages varying from food additives [1] to drug administration [2].

The continuous emergence of bacterial resistance has challenged the research community to develop novel antibiotic agents. Among the most promising of these novel antibiotic agents are metal NPs, which have shown strong antibacterial activity in an overwhelming number of studies. Generally, antibiotic-resistant bacteria appear in a relatively short period of time even when new antibiotics are released into the market. However, it is hypothesized that NPs with antibacterial activities have the potential to reduce or eliminate the evolution of more resistant bacteria because NPs target multiple biomolecules at once avoiding, the development of resistant strains.

This review summarizes and discusses proposed mechanisms of antibacterial action of different NPs. In addition, we discuss their involvement in the production of reactive oxygen species (ROS), biomolecule interaction and regulation, ATP depletion, and membrane interaction. Finally, a comprehensive analysis of the effects of NPs on the regulation of transcriptomic and proteomic profiles is discussed.

## Bacterial cell wall structure

**Fig. 1:**
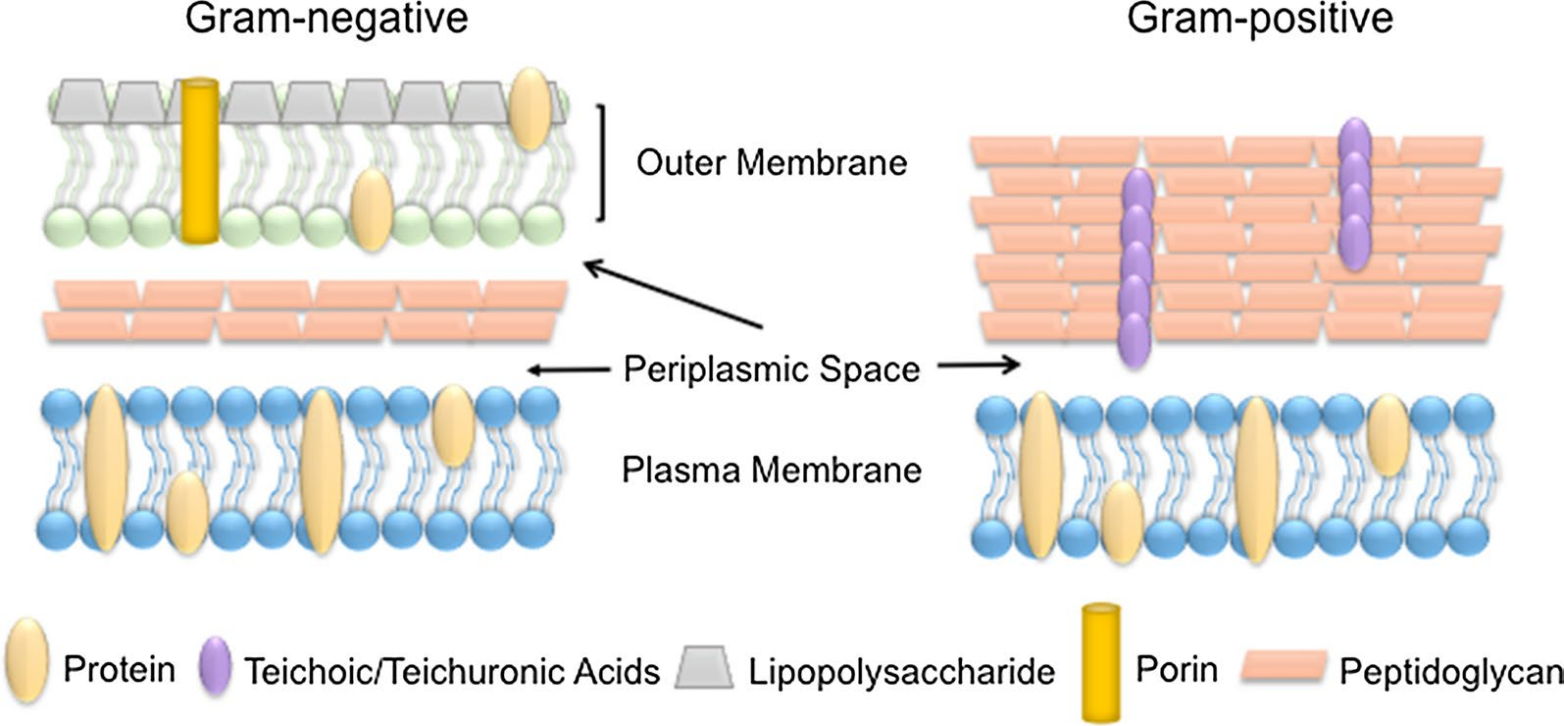
Comparison of bacterial cell wall structure

Most bacteria can be divided into two separate classifications based on their cell wall structure: Gram-positive and -negative. Gram-positive bacteria contain a thick layer of peptidoglycan in their cell walls, whereas Gram-negative bacteria have a thin peptidoglycan layer with an additional outer membrane consisting of lipopolysaccharide. This additional membrane in Gram-negative bacteria means that there is also an extra membrane layer termed periplasm (Fig. 1).

Many studies have found that Gram-positive bacteria are more resistant to NP mechanisms of action [3–7]. It is hypothesized that the differing cell walls are the reason this phenomenon exists. In the case of Gram-negative bacteria, such as *Escherichia coli*, bacterial cells are covered by a layer of lipopolysaccharides (1–3 µm thick) and peptidoglycans (~ 8 nm thick). This arrangement may facilitate the entrance of released ions from NPs into the cell. On the other hand, Gram-positive bacteria such as *Staphylococcus aureus* possess a peptidoglycan layer much thicker than Gram-negative bacteria, spanning over 80 nm with covalently attached teichoic and teichuronic acids. The cell wall destruction that occurs from physical interaction between NPs and the cell wall is more detrimental for Gram-negative bacteria as they lack the thick peptidoglycan layer found in Gram-positive bacteria that could possibly act as a protective layer.

Another potential reason for Gram-negative susceptibility to NPs is that Gram-negative bacteria are coated with lipopolysaccharide molecules, which carry a negative charge. These negatively charged molecules have a higher affinity for the positive ions that most of the NPs release, leading to a buildup and increased uptake of ions, which then cause intracellular damage.

Both Gram-positive and -negative bacteria have a negatively charged cell wall, a characteristic that is hypothesized to influence the interactions between the cell walls of the bacteria and NPs or ions released from them. Studies performed in Gram-negative bacteria such as *Salmonella typhimurium* showed that the cell wall is populated with a mosaic of anionic surfaces domains rather than a continuous layer [8]. Thus, a potential binding of a high number of NPs on these negative anionic domains may increment the focal toxicity because of the relatively high NP concentrations in these areas. Moreover, combined studies of electrophoretic mobility and mathematical calculations determined that *E. coli* is more negatively charged and rigid than *S. aureus* [9].

Changes in the electronegativity of the cell wall of bacteria can occur as a result of a change in the broth used to grow the bacteria. For example, electrophoretic mobility experiments performed in *S. typhimurium* strains grown in media with different carbon sources showed that assembly of the O-antigen on the lipopolysaccharide layer occurred when the strain was grown in a galactose-based medium, but not in a glucose-based medium. This difference in the lipopolysaccharide assembly had no effect on the electrophoretic mobility, suggesting that a change in the lipopolysaccharide entities on the cell wall as a result of a change in the electronegativity is not significant [10]. Similar observations on the electrophoretic mobility were reported when the composition of O-antigens was modified by different growth media in *E. coli* [11].

Some ROS such as hydroxyl radicals are negatively charged, meaning they cannot easily penetrate the negative cell membrane [12]. This electrostatic characteristic becomes even more important when charged capping agents are used in NP fabrication, further adding to electrostatic attraction or repulsion.

An exception to the typical influences of cell membrane charge and cell structure is heavy metal resistant bacteria. Few studies reported that these bacteria are unaffected when exposed to metallic NPs, which showed antibacterial activity against non-heavy metal resistant bacteria [13]. For example, when both Gram-negative *E. coli* and *Cupriavidus metallidurans* strains were exposed to TiO_2_, Al_2_O_3_, and carbon nanotube NPs, *E. coli* was sensitive and killed by all NPs tested, whereas *C. metallidurans* was resistant despite being also a Gram-negative bacterium, indicating that this bacterium is accustomed to being in an environment with heavy metal stress [13]. Interestingly, transmission electron microscopy analysis showed that the different types of TiO_2_-NPs (A12, A140, and R9) used in this study behaved in a different way. For example, TiO_2_ A12, which was synthesized using laser pyrrolysis [14] localized in the periplasm of both strains, whereas TiO_2_ R9 (rutile from Sigma-Aldrich, Cat # 637262) and A140 (anatase from Sigma-Aldrich, Cat # T-8141) did not, suggesting a specific mechanism of internalization. It seems that the adsorption of the NPs onto bacterial cell wall is a pre-requisite for the internalization as shown also by the periplasmic localization of Al_2_O_3_ NP in both strains.

Further studies in *C. metallidurans* have shown that the metal resistance is conferred by two large plasmids termed pMol-28 and pMol-30. pMol-28 confers resistance when the bacterium is exposed to Co^2+^, Cr^6+^, Hg^2+^ and Ni^2+^; whereas pMol-30 is activated by Ag^+^, Cd^2+^, Co^2+^, Cu^2+^, Hg^2+^, Pb^2+^, and Zn^2+^. Transcriptomic analyses showed that pMol-28 and pMol-30 induce the upregulation of 83 and 143 genes, respectively [15]; but further research is necessary to determine the function of all these upregulated genes.

The Gram-negative bacterium *Shewanella oneidensis* has similarly been shown to be able to reduce heavy metal ions when treated with CeO_2_ NPs. It was also found to be resistant to NP activity, whereas *E. coli* and *Bacillus subtilis* were sensitive [16]. In summary, it is likely that bacteria adapted to environments contaminated with heavy metals (metal stresses) are better able to cope with NP exposure either by (1) modifying the peptidoglycan layer, (2) activating genes responsible for cell wall/membrane repair, or (3) ion sequestration by metabolites or proteins (see below).

## Elements used in NP fabrication

The metals used for antimicrobial NP fabrication are almost exclusively heavy metals, which are classified as metals with a density > 5 g/cm^3^. These metals tend to be transition elements, meaning that their electron configuration is such that the d orbital of the atom is partially filled. This is important because a partially filled d orbital means that these metals are generally more redox active, facilitating the NP formation. NPs are most often formed by a “bottom up” chemical mechanism which requires a metal salt and a strong reducing agent, such as sodium borohydride [17]. The reaction involved reduces the metal cation to a neutral state, which provides a nucleation site for the metal atoms to aggregate and eventually form a NP [18].

Many transition metals perform important biological functions such as hydroxylation, redox reactions, and electron transport [19]. While these metals are essential in small quantities, they become very toxic at higher concentrations. Generally speaking, the metal cation is required for intracellular function and it must be transported into the cell. The formed NP, however, is in neutral metal and likely it cannot cross the cellular membrane. But it is known that metal NPs slowly release metal ions able to cross membranes and disrupt cellular processes from inside the cell [20].

The bactericidal activity of transition metal NPs can be attributed to many different properties, the most important being the ability to generate ROS and their affinity to associate closely with R-SH groups. The heavy metal ions of non-essential transition metals with high atomic numbers such as Ag^+^ or Hg^2+^ can easily bind to SH groups, such as in cysteine, which can directly disrupt the function of specific enzymes or break S–S bridges necessary to maintain the integrity of folded proteins, causing detrimental effects to the metabolism and physiology of the cell. The generation of ROS is particularly destructive to bacterial cells as explained later in this review.

The metal Ag has been used as an antibacterial treatment for centuries [21]. Due to its ancient use, Ag is probably the most popular element to synthesize NPs. However, many other elements have been used to fabricate NPs, including Al (Al_2_O_3_), Au, Bi, Ce, Cu (CuI, CuO, and Cu_2_O), Fe (Fe_2_O_3_), Mg (MgO), Ti (TiO_2_), and Zn (ZnO); and mixed metal oxides, antibiotic- and enzyme-conjugated NPs [22–28].

## Size, shape, and charge characteristics of NPs

Due to their small size and high surface-to-volume ratio, NPs have physical and chemical properties that differ from their bulk material. Varying the physical and chemical parameters has a profound effect on the antibacterial activity of NPs as detailed below. In Table 1 the physical and chemical characteristics of NPs discussed in this review are summarized.

**Table 1:** Physical characteristics and antibacterial activities of the literature used in this review

NP type	Size (nm)	Shape	Strain	Exposure time	Activity	Remarks/purpose	References
Ag							
	17.5	NR	*P. aeruginosa* ATCC 27317	4 h	G = 3.7 fold reduction	Citrate-capped	[7]
	38.8	NR	*S. aureus* ATCC 25923	4 h	G = 0.685 fold reduction	11-Mercaptoundecanoic-capped	
	20–25	Spherical	*A. baumanii* BAA-747, *P. aeruginosa* ATCC 27853	24 h	MIC = 0.4 µg/mL		[22]
			*B. subtilis* ATCC 6333		MIC = 1.7 µg/mL		
			*E. coli* ATCC 25922, MRSA ATCC 700698, *M. smegmatis* ATCC 700084		MIC = 0.5 µg/mL		
			*M. bovis* BCG ATCC 35374		MIC = 1.1 µg/mL		
			*S. aureus* ATCC 25923		MIC = 0.7 µg/mL		
	9–21	NR	Nitrifying bacteria	NR	EC_50_ = 0.14 µg/mL	Inhibition of nitrification	[29]
	9	Spherical	*E. coli*	24 h	IC_50_ = 6.4 µg Ag^+^/mL	Citrate-capped	[30]
	19	Spherical	*E. coli*	24 h	IC_50_ = 15.7 µg Ag^+^/mL	Citrate-capped	
	43	Spherical	*E. coli*	24 h	IC_50_ = 40.9 µg Ag^+^/mL	Citrate-capped	
	18	Spherical	*E. coli*	24 h	IC_50_ = 5.5 µg Ag^+^/mL	PVP-capped	
	23	Spherical	*E. coli*	24 h	IC_50_ = 2.2 µg Ag^+^/mL	BPEI-capped	
	9.5	Spherical	*S. mutants*	24 h	MIC = 4 µg/mL		[31]
	26				MIC = 8 µg/mL		
	79				MIC = 4 µg/mL		
	18	Spherical	*E. coli*	8 h	MIC = 50 µg/mL		[32]
	80				MIC = 200 µg/mL		
	10	Spherical	Gram-positive strains and *Bacillus*	5 d	MIC = 600 µg/L	Citrate-capped	[33]
	12				MIC = 10 µg/L	PVP-capped	
	10				MIC = 3 µg/L	BPEI-capped	
	39	Spherical	*E. coli* ATCC 10536	8 h	MIC = 50 µg/mL		[39]
	40	Triangular			MIC = 2.5 µg/mL		
	5–10	Spherical	*E. coli* MTCC 405	24 h	Z = 13 mm		[45]
			*S. aureus* MTCC 3160		Z = 10 mm		
	5–40	Spherical	*A. punctate* (lab isolate)	24 h	Z = 0 mm (at 50 µg/disc)		[46]
			*E. coli* ATCC 13534, *E. coli* ATCC 25922		Z = small (at 50 µg/disc)		
			*M. luteus* (clinical isolate)		Z = small (at 50 µg/disc)		
	142	NR	*E. coli* K12 MG 1655	1 h	100 µg/mL	Adaptive stress response	[48]
	13.5	Spherical	*E. coli* O157:H8, *S. aureus* ATCC 19636	24 h	MIC = > 3.3 nM		[50]
	5–15	Spherical	*L. monocytogenes* ISP 6508	24 h	99.9% killing at 5 wt%	Polyethylene modified	[52]
	9.2	Spherical	*E. coli* K12 MG 1655	16 h	MIC = 2 nM	Oxidized particles	[54]
	35	Amorphous	*A. vinelandii* ATCC 13705	2 days	MIC = 12 µg/mL	Carbon coated	[56]
			*N. europaea* ATCC 19718	7 days	MIC = 0.5 µg/mL		
			*P. stutzeri* ATCC 17588	1 days	MIC = 4 µg/mL		
	22.5	Spherical	*E. coli* (clinical isolate)	24 h	Z = 9–37 mm	NPs supplemented with antibiotics	[67]
			*S. aureus* (clinical isolate)		Z = 9–36 mm		
	7.1	Spherical	*E. coli* MTCC 062	18 h	MIC = 3.6 µg/mL		[69]
			*P. aeruginosa* MTCC 424		MIC = 2.7 µg/mL		
	142	Spherical	*E. coli* K12 MG 1655	10 min	140 µg/mL	Transcriptome analysis	[70]
	35.4	Spherical	*E. coli* K12 ATCC 25404	6 h	97.7% killing at 0.32 µg/mL	Anaerobically produced	[83]
		Irregular			99.8% killing at 0.32 µg/mL	Aerobically produced	
	30 nm		*E. coli*	1 d	100 µg/mL	Protein-binding silver studies	[85]
	60		*E. coli* K12 MG 1655	2 h	1, 10, 50 µg/mL	Gene expression studies	[87]
	20–30	Spherical	*P.* ssp FPC 951		200 µg/mL	Stress response studies	[88]
Bio-Ag	2–10	NR	*K. pneumonia* ATCC 700603	24 h	Z = 2 mm at 100 µg/mL	Synthesized from *Actinobacteria* CGG 11n supernatant	[65]
			*P. mirabilis* (collection), *S. infantis* (collection)		Z = 0 mm at 100 µg/mL		
			*P. aeruginosa* ATCC 10145		Z = 10 mm at 100 µg/mL		
			*S. aureus* ATCC 6338		Z = 8 mm at 100 µg/mL		
Ag/CeO_2_		Rod	*E. coli* ATCC 8099	2 h	G = ~ threefold reduction (100 µg/mL)	Used 1% wt%	[38]
		Cube			G = fourfold reduction (100 µg/mL)		
		Particles			G = ~ 3.5 fold reduction (100 µg/mL)		
		Rod			G = threefold reduction (100 µg/mL)	Used 2% wt%	
		Cube			G = ~ fourfold reduction (100 µg/mL)		
		Particles			G = ~ fourfold reduction (100 µg/mL)		
Al_2_O_3_	11	Spherical	*E. coli* MG 1655	24 h	MIC = 106 µg/mL		[13]
Au	8.4	Spherical	*A. baumannii*, *E*. *coli* J96, *E. coli* O157:H7, MRSA, *P. aeruginosa*, PDRAB, *S. aureus*	9 h	MIC = 8 µg/mL	Coupled to vancomycin	[66]
			*E. faecalis*, *E. faecium*, *E. faecalis* VRE1		MIC = 16 µg/mL		
			*E. faecium* VRE4		MIC = 32 µg/mL		
	50, 100		*S. oneidensis* MR-1			COOH^−^, quaternary amine NMe_3_^+^), and methyl-conjugated (CH_3_–) NP attachment study	[71]
CeO_2_	6	Square	*B. subtilis* ATCC 6333 *E. coli* ATCC 700926	24 h	Z = ~ 3.3 mm Z = ~ 0.2 mm		[16]
	15	Circular, ovoid	*B. subtilis* ATCC 6333 *E. coli* ATCC 700926	24 h	Z = ~ 0.3 mm Z = ~ 3.3 mm		
	22	Ovoid, rectangular, triangular	*B. subtilis* ATCC 6333 *E. coli* ATCC 700926	24 h	Z = ~ 2.2 mm Z = ~ 1.8 mm		
	40	Heterogeneous	*B. subtilis* ATCC 6333 *E. coli* ATCC 700926	24 h	Z = ~ 3 mm Z = ~ 1.0 mm		
	7	Ellipsoidal	*E. coli* RR1	3 h	MIC = 500 µg/mL		[36]
	2–4	Spherical	*L. monocytogenes* ISP 6508	24 h	99.9% killing at 5 wt%	Polyethylene modified	[52]
	7	NR	*E. coli* RR1	3 h	MIC = 500 µg/mL		[36]
Cu_2_O	40	Heterogeneous	*E. coli*	18 h	MBC = 0.1 mM	Tryptophan-capped	[79]
CuO	22.4–94.8	Equi-axes	*S. aureus* EMRSA-16, *S. aureus* (MRSA) 252	4 h	MBC = 1000 µg/mL		[49]
			*S. aureus* EMRSA-15, *E. coli* NCTC 9001		MBC = 250 µg/mL		
			*S. aureus* NCTC 6571		MBC = 100 µg/mL		
			*S. aureus* ‘Golden’ (lab isolate), *S. epidermidis* SE-4 and SE-51		MBC = 2500 µg/mL		
			*P. aeruginosa* PAOI, *Proteus* spp. (lab isolate)		MBC = 5000 µg/mL		
	30	Heterogeneous	*E. coli*	18 h	MBC = 0.25 mM	Tryptophan-capped	[79]
MgO	4	Square, polyhedral	*E. coli* C3000, *B. megaterium* ATCC 14581	1 h	NG at 250 mg	Agar overlay with aerogel	[41]
			*B. subtilis* ATCC 6333		48% killed		
	20	Amorphous	*E. coli* XL-1 blue			Metabolic pathway regulation study	[68]
Mg(OH)_2_- MgCl_2_	12.9	Flake	*E. coli*	NR	88% killed at 100 µg/mL	Co-precipitated with MgCl_2_	[43]
Mg(OH)_2_- MgSO_4_	21.4	Sheet			60% killed at 300 µg/mL	Co-precipitated with MgSO_4_	
Mg(OH)_2_- MgO	44.8	Plate			53% killed at 500 µg/mL	Co-precipitated with MgO	
TiO_2_	12	Spherical	*E. coli* MG 1655	24 h	MIC = 100 µg/mL		[13]
	17	Spherical	*E. coli* MG 1655	24 h	MIC = 100 µg/mL		
	21	Spherical	*E. coli* MG 1655	24 h	MIC = 100 µg/mL		
	25	Spherical	*E. coli* MG 1655	24 h	MIC = 100 µg/mL		
	< 100	Elongated	*E. coli* MG 1655	24 h	MIC = 100 µg/mL		
	250–300	Elongated	*A. baumanii* BAA-747, *P. aeruginosa* ATCC 27853	24 h	MIC = 20 µg/mL		
			*B. subtilis* ATCC 6333, MRSA ATCC 700698, *S. aureus* ATCC 25923		MIC = 54 µg/mL		
			*E. coli* ATCC 25922		MIC = 59 µg/mL		
			*M. bovis* BCG ATCC 35374		MIC = 11 µg/mL		
			*M. smegmatis* ATCC 700084		MIC = 5 µg/mL		
	23	NR	*E. coli* MG 1655	5 h	MIC = 10 µg/mL	Transcriptomic and proteomic analyses	[34]
	10	NR	*E. coli*		LC_50_ = 14.2 µg/mL	Sulfur-coated	[82]
	3.8				LC_50_ = > 1000 µg/mL	Nitrogen-fluorine co-doped	
	NR				LC_50_ = 2.2 µg/mL	Commercial P25 (Degussa)	
	NR				LC_50_ = 2.6 µg/mL	Commercial Sigma	
	10		*E. coli* K12 MG 1655	2 h	1, 10, 50 µg/mL	Gene expression studies	[87]
ZnO	12	Spherical	*E. coli*	24 h	Z = 31 mm	Thiol-capped	[12]
	19	Sphere-like	*E. coli*	3 h	MIC = 50 µg/mL		[84]

G, growth; LC_50_, lethal concentration; MBC, minimal bactericidal concentration; MIC, minimal inhibitory concentration; MRSA, methicillin-resistant *S. aureus*; NG, no growth; NR, not reported; PDRAB, pandrug-resistant *A. baumannii*; Z, zone of inhibition

Typically, smaller NPs have higher antibacterial activity [12, 13, 22, 29–32]. However, some studies have shown that larger NPs are more effective, indicating that size alone is not the most important factor of their toxicity [33, 34]. Other factors can include the formulation process, the environment, the bacterial defense mechanism, and the physical characteristics of the NP.

The fact that small NPs tend to be more toxic than large NPs can be explained by the small NPs relative larger surface area to volume ratio as compared to larger NPs. This can greatly increase the production of ROS is greatly increased (see below), which consequently can damage and inactivate essential biomolecules, including DNA, proteins, and lipids [35].

NPs are hypothesized to be able to participate in sub-cellular reactions as their size is comparable to biological molecules, i.e., large protein complexes [36]. Having characteristics differing from larger materials due to their size and surface chemistry, NPs have shown an ability to inhibit the growth of bacteria and consequently have been used as a tool to combat infectious disease [37]. Even with promising results being observed, there is a debate as to how this inhibition occurs and what mechanisms are involved.

For NPs the most common shape is spherical, although other shapes such sheets, plates, tubes, cubes, rods, and triangles have also been reported. Nanocubes and rods (CeO_2_-NPs) seem to be more effective than other shapes, possibly due to the exposed planes and to the oxidation levels of the metals [38]. This explanation was supported by the analysis of the exposed crystal facets, which suggested that less stable planes require less energy to form oxygen vacancies, linking the bactericidal activity of the NPs to the stability of the planes [38]. Even amongst NPs with identical surface areas, the shape is important as the planes with high atom density facets increase reactivity [39, 40].

When dissecting the nanostructure of a NP, there is a correlation between the presence of corners, edges, or defects (increased abrasiveness) and an increase in the toxicity, potentially because (i) the increased area helps in the adsorption and binding of compounds or (ii) the increase in surface defects also increases the surface area to volume ratio which has a direct effect on ROS generation [12, 16, 41].

Physical deformations also increase mechanical damage. For instance, ZnO-NPs with defects can be activated by UV and visible light, creating electron hole pairs resulting in the splitting of suspended H_2_O molecules into OH^−^ and H^+^. The dissolved molecules eventually react to form H_2_O_2_, a ROS that is able to penetrate the cell membrane and kill bacteria. This phenomenon has also been observed in *E. coli* treated with Ag-NPs [12]. However, other studies reported that the crystalline phase of TiO_2_ does not affect toxicity. For example, the two crystalline forms of TiO_2_ rutile and anatase were assayed with no significant differences in their antibacterial activity [13]. In the same study, single- and multi-walled carbon nanotubes were also tested, and authors concluded that impurities in the formulation did not affect their toxicity. They hypothesized that this observation is likely due to the fact that impurities could be inside of the tubes, in an area that does not interact with the cell membrane. In addition, they found that single-walled carbon nanotubes were more toxic than their multi-walled counterparts, suggesting that diameter may play a role in toxicity [13].

**Fig. 2:**
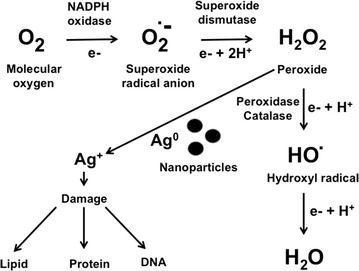
Scheme describing the role of NPs in the generation of ROS

Another important factor in antibacterial activity is the charge of the NP. Positively charged NPs, such as amino-functionalized polystyrene particles, were able to alter the function of the electron transport chain in bacteria [30]. A more detailed study using an *E. coli* single gene deletion library identified that bacteria with mutations on ubiquinone biosynthesis related genes were more sensitive when exposed to the positively charged NPs [42]. Ubiquinone or coenzyme Q_10_ is a component of the electron transport chain and is essential for the aerobic respiration. Authors concluded that the exposure of the bacteria to these NPs generates ROS that induces oxidative stress (Fig. 2), which is consequently quenched either by a direct interaction with ubiquinone or by its function in the electron transport chain [42].

More importantly, a positive charge in the NPs has been shown to enhance toxicity because the negative charge of the bacterial cell wall electrostatically attracts the positively charged NPs, causing them to be more effective [30, 33, 41, 43]. For example, a disruption in the cell wall was observed by electron microscopy when *B. subtilis* cells were exposed to MgO-NPs [41], suggesting that the desiccant nature of this oxide could contribute to its killing activity.

Acidic conditions have been found to favor binding of the NPs to the bacterial wall, supporting the fact that electrostatic interactions play an important role in this process [44]. Positively charged Ag-polyethylenimine (BPEI)-NPs tightly adhere to the bacterial surface, some even fusing with the cell wall, while no attachment has been observed for the negatively charged citrate-Ag-NPs [30]. Finally, the Ag-BPEI-NPs induced a response similar to any cationic particle signifying that bactericidal activity is the most important contributor to the charge [30].

## Effect of capping agents and halogen treatment on antibacterial activity

During NP fabrication, a capping agent is added to increase the stability and facilitate the dispersion of the NPs. These agents may have a direct effect on the toxicity of the NPs, likely due to their ability to reduce NP agglomeration [6, 7, 12, 45, 46]. When comparing Ag-NPs with Ag-NPs stabilized with citrate, chitosan, or polyvinyl acetate (PVA); citrate- and chitosan-capped Ag-NPs are most effective in the killing of bacteria, probably because of an accelerated generation of Ag^+^ from these NPs [6]. The capping agent chitosan has been shown to possess antibacterial activities against *E. coli*, but in concentrations > 200 ppm, suggesting that the antibacterial activities of chitosan-capped Ag-NPs is not related to this polysaccharide [47], However, when comparing citrate-capped vs. 11-mercaptoundecanoic acid-capped Ag-NPs, the 11-mercaptoundecanoic acid-capped Ag-NPs are more toxic as a result of an agglomeration of these NPs on the cell wall of the bacterium [7]. It should be stressed that the experiments were performed in *P. aeruginosa* which have a hydrophilic cell wall. Other studies have also reported that citrate-capped Ag-NPs are less toxic [33] when comparing citrate-capped Ag-NPs (10 nm) to uncoated H_2_–Ag-NPs (18 nm), polyvinyl pyrrolidone (PVP)–Ag-NPs (12 nm), and Ag-BPEI-NPs (10 nm) [30].

As a result of the toxicity generated by the chemical compounds used for NP fabrication, green technologies were developed to overcome this issue. The presence of reducing compounds in plant extracts have led to their increased usage over the last few years. Furthermore, functional groups can be added to the surface of the NPs. For example, the morphology of Ag-NPs changes depending on the stabilizer used [45]. Using a UV–Vis absorption peak, it was discovered that increasing the concentration of plant extract leads to a stronger binding of the capping agents and the biomolecules. Ultimately, the study concluded that the positively charged detergent cetyl trimethylammonium bromide (CTAB) enhances NP toxicity by directing the adsorption on specific crystal planes of the NPs. Moreover, an aggregation process that occurs between the negatively charged cell wall and the presence of CTAB has been proposed, suggesting a synergistic effect between the CTAB and NPs [39].

Treating NPs with halogens can increase their antibacterial activity [41]. For instance, a formulation of NPs using an aerogel was prepared with MgO and Cl_2_ or Br_2_ to solve the problem of the high toxicity and vapor pressure associated with halogens [41]. The aerogel formation meant that Cl_2_ was converted into a dry powder form with no loss of activity. The resulting NPs were equally active against both Gram-negative and -positive bacteria and even had slight activity against endospores. Authors concluded that the high activity was likely due to the abrasiveness, high surface area, and oxidizing power of the halogen [41].

## Ion release from NPs

NPs are constantly undergoing dissolution because of the electrochemical potential in solution. It has been shown that the antibacterial activity of NPs is based on and proportional to the release of ions, although other mechanisms can be involved as well [30, 38, 40, 48–50]. The concentration of NPs directly effects toxicity because a larger concentration of NPs releases more ions [51, 52] with a concomitant increase over time [53], correlating with findings that longer incubation time decrease viability.

It has been found that *E. coli* cells treated with Al_2_O_3_- and TiO_2_-NPs were more impacted by Al_2_O_3_, with a lower concentration of Al_2_O_3_ required to have a similar antibacterial activity as TiO_2_ [13]. Using inductively coupled plasma mass spectrometry, it was found that Al_2_O_3_ contained 0.3% Al^3+^ while there was no Ti^4+^ in the TiO_2_ formulation, suggesting that ion release may play a role in toxicity [13]. Additionally, when Ag-NP impurities are removed there was a dramatic reduction in their toxicity, likely due to removal of leached Ag^+^ from the NPs into the solution, suggesting that ion release alters toxicity [33].

Ions are often responsible for toxicity. When metal ions in solution are exposed to bacterial cell is, they become uniformly distributed in the environment surrounding the bacterial cell with no specific localization. In contrast, NPs that interact with the bacterial cell wall produce a focal source of ions continuously release ions, and causing more toxicity to the cells [48]. The large generated ion concentration further helps to penetrate the cells. As a consequence, the NP dissolution is localized around the bacterial cell membrane, with the kinetic of dissolution depending on the size and shape of the NP. The surface morphology of the NPs have a profound effect on the activity of the NPs and when the surface of the NPs are rougher, the dissolution occurs faster [50]. Additionally, the larger surface area to volume ratio in smaller NPs results in faster dissolution.

NPs have higher antibacterial activity than their bulk counterparts [12, 51–55]. While antibacterial activity is evident from ions alone, the fact that NPs are more toxic indicates that other mechanisms contribute to toxicity. However, contradictory evidence has been reported. For instance, Ag^+^ was 20–48 times more toxic than Ag-NPs, but their viability tests were done specifically on nitrogen-cycling bacteria and other factors/mechanisms might be involved as well [56].

The release of ions from NPs appears to be element dependent. For example, Cu-NPs released 253 × more ions than Ag-NPs, producing higher antibacterial activity, possibly due to Cu’s higher oxidation susceptibility [52]. To attain the same toxicity level as a fixed concentration of Cu-NPs would thus require an increased amount of Ag-NPs is necessary to attain the same toxicity level as a fixed concentration of Cu-NPs, consistent with the idea that ion release is crucial for antibacterial activity. However, Ag-NPs are more efficient, meaning that although significantly fewer ions are released, the antibacterial activity produced by the same number of Ag^+^ is much higher than produced by the same number of Cu^2+^ [52].

The fact that Ag-NPs are still more efficient to kill bacteria than Cu-NPs (regardless the ion generation), can be explained by the essentiality of Cu in physiological systems. Cu is an essential element playing a role as a co-factor for different enzymatic systems, such as those involved in redox reactions essential to cellular respiration (cytochrome oxidase) and superoxide dismutase (antioxidant defense) [57]. Thus, the differences in the antimicrobial potency of Ag^+^ and Cu^2+^ can be explained by the following hypotheses: (1) both Ag^+^ and Cu^2+^ have a high affinity for thiols, including cysteine, the unique thiol-containing amino acid. Cu^2+^ has a higher affinity (× 100) to cysteine as compared to Ag^+^ [58]. However, Cu^2+^ undergoes a mechanism of homeostasis when binding cysteine. For instance, when Cu^2+^ binds cysteine it is reduced to Cu^+^ with a concomitant production of cystine, the oxidized dimer of cysteine, following a dismutation of the displaced Cu^+^ to regenerate Cu^2+^ [59]. In the case of Ag^+^, once it binds the cysteine residue, there is no homeostasis mechanism and the metal precipitates on the cysteine, leaving this residue unavailable as a functional amino acid. (2) Biomolecules such as reduced glutathione (GSH) can undergo oxidation as a result of Cu-catalyzed reaction [60]. GSH can coordinate Cu^2+^ with high affinity as well as other bacterial proteins, such as the cysteine-rich metallothioneines. These proteins possess an unusual number of cysteine residues in their sequence and probably have a role in toxicity defense against metals [61]. Ultimately, Cu^2+^ binding to cysteines will follow the homeostasis mechanism explained in (1), whereas Ag^+^ will bind irreversibly to cysteines. (3) Bacterial cells possess Cu efflux pumps, such as the *E. coli* CopA, a P-type Cu^+^ efflux ATPase, which maintains a low intracellular concentration of Cu [62]. Other Cu-binding proteins are the CueO multi-Cu oxidase [63] and the CusCFBA multicomponent efflux transport system [64], both contributing to the intracellular homeostasis of Cu and protection of the bacterial cell.

Taking all this information into account, the fact that more Cu^2+^ ions are necessary to reach the same antibacterial activity as Ag^+^ is based on the fact that Cu is an essential element and cells possess mechanisms to maintain its homeostasis by avoiding its intracellular toxicity. On the other hand, Ag^+^ is not an essential element and by irreversibly binding the cysteines, it can poison vital enzymatic systems, such as the main energy source of the cells or the respiratory electron transport chains.

## Resistance to antibiotics

**Fig. 3:**
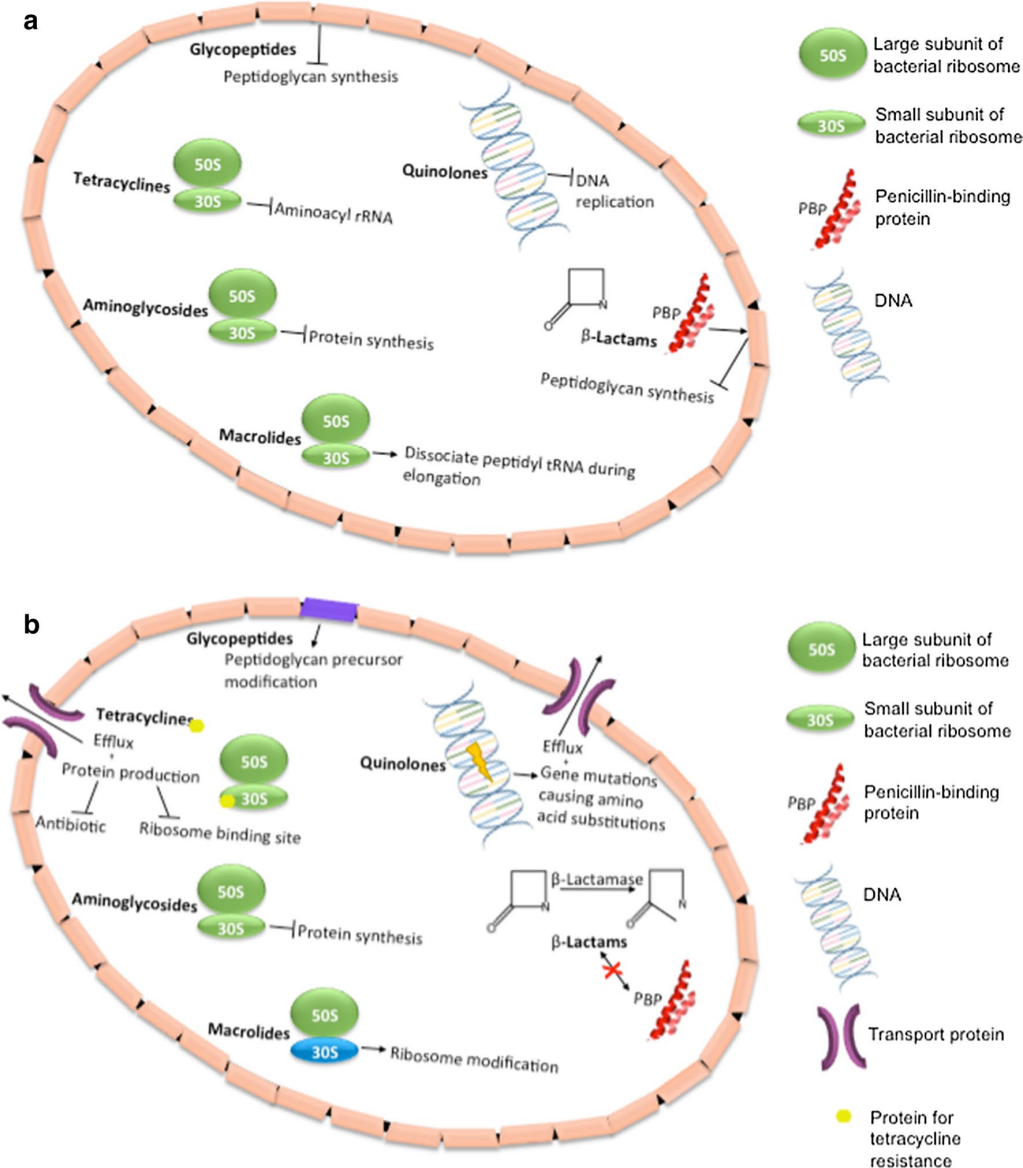
Mechanisms of selected antibiotic classes and antibacterial resistance. **a** Illustration describing the antibiotic mechanisms of β-lactams (e.g. penicillin, carbapenems, cephalosporins), aminoglycosides (e.g. amikacin, kanamycin, gentamicin), glycopeptides (e.g. vancomycin, teicoplanin, decaplanin), macrolides (e.g. azithromycin, erythromycin, clarithromycin), tetracyclines (e.g. tetracycline, doxycycline, minocycline), and quinolones (e.g. ciprofloxacin, levofloxacin, moxifloxacin). **b** Mechanisms of antibiotic resistance develop by bacteria

Microbes have developed many systems to neutralize antibiotics. We describe, as an example, a few of the mechanisms of resistance to antibiotics in bacteria, which may potentially be relevant to NP resistance (Fig. 3a, b). About 60–70% of the current antibiotics are not effective against intracellular infections due to their low intracellular retention as a result of their poor permeability. The hydrophilic nature of common antibiotics like beta-lactams and aminoglycoside makes cell penetration difficult. NPs represent an attractive solution for the hydrophilicity barrier because they can often penetrate cells, especially in phagocytic cells (macrophages), which may engulf NPs and increase their intracellular activity [44].

Aminoglycoside antibiotics diffuse through porin channels of Gram-negative bacteria and are then actively transported into the cell where they irreversibly bind to the 30S ribosomal subunit, inhibiting protein synthesis [65]. On the other hand, beta-lactam antibiotics attach to penicillin-binding proteins and ultimately inhibit cell wall peptidoglycan synthesis and inactivate autolytic enzyme inhibitors [65]. Because this class of antibiotic facilitates a breakdown of the cell wall, it is possible that NPs are more effective combined with antibiotics simply because it is easier for the NPs to enter the cell. The reverse is true as well, when NPs disintegrate the cell wall, it is easier for antibiotics to enter the cell, especially aminoglycosides whose mechanism of action does not involve cell wall breakdown. Both aminoglycosides and beta-lactam antibiotics contain hydroxyl and amino groups that could interact as targets of the NPs [65]. It is worth noting that NPs have not been show to undergo a morphological change with the addition of antibiotics [44].

Antibiotic-conjugated NPs exhibit a higher antibacterial activity than the antibiotic alone or NP alone, indicating a synergistic effect and hinting that NPs and antibiotics use different antibacterial mechanisms [44, 65, 66]. In addition, a study using *E. coli* and *S. aureus* in combination with penicillin G, amoxicillin, erythromycin, clindamycin, or vancomycin found that the presence of Ag-NPs increased efficacy of the antibiotics, without any no intended conjugation [67]. However, an unintentional binding may still have occurred between NP and antibiotic [65].

## Bacterial cell wall interactions and cell penetration

The exposure of NPs to bacterial cells can lead to membrane damage caused by NP adsorption sometimes followed by penetration into the cell [16, 36, 41, 48]. Many studies suggest that adsorption on the cell wall following its disintegration is the primary mechanism of toxicity [13, 36, 48, 68]. Adsorption of NPs leads to cell wall depolarization, which changes the typically negative charge of the wall to become more permeable. It has been reported that the bacterial cell wall become blurry, indicating cell wall degradation as shown by a laser scanning confocal microscope [5]. In this study, authors suggested a bimodal mechanism of action of Ag-NPs. In the first step, the cell wall is destroyed with subsequent penetration of NPs. In a second step, ROS are formed that inhibit ATP production and DNA replication. Since the production of ROS has been shown to counteract the cell built-in antioxidant defense and lead to cell wall into the cell damage, it is possible that the production of ROS plays a part in the primary step as well [69].

Ag-NPs themselves have also been found to associate with the cell wall [48, 54]. This is hypothesized to be a source of toxicity as this association can result in degradation, allowing ions to enter into the cytosol. Ag-NPs also have an ability to cause irregular pit formations on the cell wall [39, 47], which facilitate ions entering the cell and halts transport regulation as observed by transmission electron microscopy. Moreover, it has been hypothesized that Ag^+^ may enter the cell through cation selective porins, which provide another possible mechanism for Ag^+^ to enter the cell and cause toxicity [70].

Criticism has been raised regarding the current bacterial cell analysis methods due to the common assumption that the cell surface is uniform with all embedded molecules having a totipotent binding affinity, as well as the assumption that all cells in a population have the same surface tension [71]. This assumption was supported by challenging the assumption of uniformity by binding Au-NPs to *S. oneidensis* bacteria for the study of spatial heterogeneity. It was found that carboxylic acid functionalized NPs exhibited a preferential attachment to the subpolar area of the cell. When a mutant lacking type IV pili proteins was substituted, there was no longer a binding preference [71].

Contrary to many findings of cell permeation, the interaction of MgO-NPs with the cell wall is the main source of toxicity to bacteria even though no cell penetration occurs [68]. Similarly, Mg(OH)_2_-NPs electrostatically adsorb onto the bacterial cell wall and destroy the cell wall with no NP penetration into the cell, but NP aggregation has been observed on the cell surface [43]. Similar studies have reported that when NPs interacts with the bacterial cell wall, penetration does not always occur [16, 28]. Even when toxic NPs adsorb onto the surface and enter the periplasmic space, internalization is not always toxic [13], which signifies that aggregation may constitute a significant source of toxicity.

Extracellular NP aggregation has been observed in numerous studies, sometimes with NPs aggregating together and sometimes with NPs aggregating with bacterial cells [12, 13, 16, 36, 41, 47, 52, 72]. The aggregation can lead to cell envelope damage and changes in the cell of smoothness and thickness [41]. However, it has been reported that capping ZnO-NPs with thiol prevented clumping, suggesting that capping is a potential solution for the aggregation issues [12]. NP aggregation can also be a serious problem because if the NPs are aggregating with one another, interaction with the bacterial cell wall is prevented, inhibiting toxic activity [13].

NP aggregation can be predicted from the measurement of zeta potential, which indicates the stability of colloidal suspensions [73]. A largely positive or largely negative zeta potential generally means that the colloidal suspension is highly stable (very low aggregation) with the optimal potential being > 30 or < − 30 mV. Even at the optimal zeta potential, NPs can still aggregate with each other as a result of protein complexation. In this regard, the thermodynamics of protein-NP complexation was investigated [74] using different sizes of Au-NPs and proteins, such as green fluorescent protein (a beta barrel protein) [75], BSA (a triangular prismatic protein) [76], and PhosA (an orthorhombic shaped protein) [77]. The study reported that proteins bind to the Au-NPs in different ratios. For example, GFP- and PhosA-NP had a ratio of approximately 4:1 and 1:2.5 (protein:NP), respectively, whereas in the case of BSA, a ratio < 2:1 (protein:NP) induces the complexation [74]. Consequently, a complex formation between protein and NPs is independent of the aggregation induced by the zeta potential and may govern the aggregation of NPs on the cell wall of the bacteria.

## Cellular leakage

When the bacterial strains *E. coli* and *S. aureus* were treated with Ag^+^, both strains underwent lysis [78]. The damaged cells were viewed using transmission electron microscopy imaging and it was revealed that the cell wall had physically separated from the internal cellular environment and that electron dense aggregation of compounds were surrounding the lysed cell. The observed compounds may have been the result of the interaction between Ag^+^ with negatively charged compounds located in the bacterial cell wall such as phosphate, carboxyl, and amino groups; causing Ag precipitation [33].

Ag-NPs are able to create a barrier between the cell wall and the cytoplasm more effectively in Gram-negative *E. coli* than in Gram-positive *S. aureus*, indicating that perhaps the thick peptidoglycan layer present in Gram-positive bacteria plays a role in protecting the cell from NP impregnation, but only at specific NP concentrations [33, 50, 78]. Further studies treated *E. coli* and *S. aureus* with Ag^+^ and a separation of the cell membrane from the cell wall was observed in both strains, as well as, electron dense granules surrounding the cells [3, 78]. Similar results were reported when both Ag- and Cu-NPs were tested against the Gram-positive *L. monocytogenes* [52]. This phenomenon is known to happen during plasmolysis, the process of a cell losing water and it has been hypothesized that this may occur due to cell wall destabilization causing a release of ions internally [52].

Several microscopic techniques were used to uncover changes in structural/mechanical properties of the cell wall surface upon Ag-NP exposure and the consequent destruction of the bacterial cell membrane [79]. Ag-NP exposure was found to reduce cell membrane integrity with an increase in the permeability, likely due to the neutralization of the cell membrane surface charge. When *E. coli* cells were exposed to Ag-NPs it was found to have a different surface morphology compared to untreated controls. Microscopic imaging showed that treated cells had disrupted membranes with intracellular components pooling around the cells due to membrane leakage. Similar results of lost cell integrity and appearance of cellular debris outside of the cell were observed when *P. aeruginosa* cells were treated with Ag-NP. Interestingly, an elongation of the cells was also observed, possibly due to stress conditions arresting cell division. Moreover, *E. coli* cells suffered rupture of the cell wall upon Ag-NP and even Ag^+^ exposure, eventually developing an electrostatic imbalance, collapsing the proton motive force, leading to a leakage of intracellular K^+^, and depleting almost the entire cell’s supply of K^+^ in a period of time as short as 5 min [55]. Other NPs such as ZnO-NPs [69], Aerogel-MgO-NPs [41], and TiO_2_-NPs [34] have been reported to also cause a loss of membrane integrity and leakage.

## Reactive oxygen species

ROS are species of oxygen that are highly reactive and are produced during basic metabolism. Universal intracellular mechanisms of defense have evolved to cope with this undesired chemical to avoid damage to essential biomolecules in the cell. However, under high levels of stress, the levels of ROS can increase significantly and it is hypothesized that their generation is one of the focal NP mechanisms of action that inhibit bacterial growth [12, 30, 68, 72, 80]. ROS are produced when oxygen enters undesired reduction states and transforms into free radicals, superoxides, and peroxides, rather than water. A stress on the cell, such as UV light, DNA damage, and NPs, can cause ROS production to increase to a level that is toxic to the cell [81], and can cause cell damage or cell death [81].

NPs have been shown to generate free radicals with an increase in NP concentration leading to a concomitant increase of ROS [29, 34, 50, 69, 82]. Even *C. metallidurans*, a bacteria adapted to heavy metal stress, undergoes a ROS increase during NP exposure [13].

The increasing levels of NPs in the environment may cause a perturbation to the native bacterial populations, such as nitrifying bacteria with essential roles in the transformation of ammonia to nitrates in municipal sewage treatment. It has been shown that although ROS are produced when nitrifying bacteria were exposed to Ag-NP and AgCl colloids, Ag^+^ ions were responsible for bacterial growth inhibition [29], which can be explained by the Ag concentration. When nitrogen-cycling bacteria are exposed to sublethal concentrations of Ag-NPs nitrifying genes are upregulated, however, upon exposure to higher concentrations of Ag-NPs, the upregulation stimulus is no longer present [83]. It is possible that at high concentrations of NPs, loss of cellular integrity interferes with the generation of ROS.

The oxidation state of the metal in the NPs may contribute to the bactericidal effect. For example, Cu_2_O-NPs have higher antibacterial activity than CuO-NPs, indicating that oxidation could play a role in toxicity [79]. When O_2_ is consumed to react with Cu_2_O and form Cu^2+^, this cation may react with superoxide (O_2_^−^), leading to sustained oxidative stress. These superoxide molecules may reduce Cu^2+^ to Cu^+^ and in turn generate H_2_O_2_, which can react with Cu again making OH^−^. Higher concentrations of OH^−^ have been measured in cells which have been exposed to CuO-NPs than Cu_2_O-NPs, however intracellular proteins tend to interact more with Cu_2_O than CuO [79].

### ROS and the cell membrane

Both intracellular and extracellular ROS are able to disrupt cell membranes [38]. One way of alteration of the cell membrane is by lipid oxidation which can easily be generated by free radicals [50]. Interestingly, in the case of *S. aureus*, lipids were not as affected as expected, probably due to the thicker cell wall structure of Gram-positive bacteria. Some ROS such as OH radicals are negatively charged, meaning that they cannot easily penetrate the negative charged cell membrane [12], regardless of Gram classification. However, H_2_O_2_ is a commonly produced ROS which is able to penetrate the cell membrane and kill bacteria [12].

ROS formation at the cell wall is due to positive NPs interacting with the negative charge on the cell wall [30]. Damage is further increased by the production of ROS, which has been shown to counteract the antioxidant defense built into the cell by surpassing its capacity, damaging the cell membrane [68]. Some studies found that free radicals are able to induce cellular membrane damage [50] and the oxidative stress can lead to lipid peroxidation, inhibiting bacterial growth [71, 81].

Ag-NPs interrupt the cellular respiration process [40], releasing Ag^+^ ions that preferentially inhibit the site between b-cytochromes and cytochrome α2 in the respiratory chain process [80]. Although the Ag^+^ ions are responsible for inhibiting the site and increasing ROS, Ag-NPs have been shown to produce more ROS than Ag^+^ ions alone [29]. Surprisingly, ROS are able to damage cellular DNA without visible membrane damage, suggesting a complex mechanism of toxicity [29]. Interestingly, Ag^+^ ions have no differences between aerobic and anaerobic conditions, meaning that oxidative stress is not a crucial toxicity mechanism [83].

It is not clear yet whether oxidative stress is the primary or secondary mechanism of killing. For example, exposure of Ag–CeO_2_-NPs to *E. coli* generated OH^−^, H_2_O_2_, and O_2_^−^. The Ag^+^ toxicity was insignificant in comparison to the harmful ROS production because while Ag^+^ helped generate intracellular ROS, which disrupted the cell wall and membrane, the extracellular ROS continued the production of intracellular ROS and was ultimately responsible for cell inactivation. H_2_O_2_ specifically largely contributed to the antibacterial activity [38], suggesting that catalytic oxidation is the main mechanism in the bactericidal process. However, another study found that oxidative stress is a secondary mechanism in the bacterial killing process [83].

### The antioxidant glutathione

Oxidative stress can lead to and increased depletion of GSH [71, 81]. The intracellular ROS production in Gram-negative bacteria can be measured by detecting the ratio of GSH to oxidize glutathione (GSSG). GSH is a tripeptide thiol, which reduces disulfide bonds to cysteines with a concomitant oxidation to GSSG. This reaction protects the cell from harmful redox reactions by scavenging ROS molecules [69]. For example, exposure of bacteria to Ag-NPs led to a GSH depletion with an increase in the formation of GSSG [69]. Similar results were obtained when ZnO- and TiO_2_- NPs were exposed to *E. coli* [72].

## Interaction of NPs with intra/extracellular compounds and DNA

It is hypothesized that NP concentration decreases as the NPs interact and bind with organic materials in the culture broth and damaged cell components [30]. For example, it has been reported that ZnO-NP toxicity changed dramatically depending on the media in which they were suspended, suggesting that a complexation between Zn^2+^ and specific molecules of the broth occurs with a reduction in the antibacterial toxicity [84]. Media components that may interfere include sodium citrate, phosphates that form Zn_3_(PO_4_)_2_, amino acid, and peptides [84].

Other ligands are also able to react with Ag^+^ and Ag-NPs, decreasing antibacterial activity due to decreased availability as demonstrated by their binding to Cl^−^, S^2−^, cysteines and phosphates, which are abundant in aquatic environments [84]. Moreover, bacteria treated with CuO- and Ag-NP showed that bacterial secretion of exopolysaccharides interacted with the NPs, extracellularly trapping the NPs and decreasing toxicity [50, 84].

NPs sized between 1–12 nm seem to be able to penetrate into the bacterial intracellular environment [38, 39]. Once inside the cell, the NPs release ions, which target multiple sites simultaneously. Ag-NPs are commonly used to investigate protein-binding properties due to their affinity for thiol groups [5, 47]. Based on a proteomic study, it has been shown that approximately 65% of *E. coli* proteins bound to Ag-NPs are enzymes [85]. Amongst the enzymes with a similar high affinity for Ag-NPs are tryptophanase, alcohol dehydrogenase, and cytochrome C, as demonstrated in a time-dependent reaction, suggesting a hierarchical binding to proteins [85]. The non-enzymatic proteins that Ag-NPs bind to are involved in membrane integrity, such as membrane porins (OmpA and OmpB), chaperonins, and periplasmic peptide binding proteins [85]. Porin binding could potentially alter the passive porin channel structure to allow small NPs to enter as it has been shown that NPs smaller than 10 nm in diameter could be passing through porins [85]. The high affinity of the periplasmic peptide binding protein towards Ag-NPs may explain why these NPs accumulate in the periplasmic area of the bacteria [29].

As mentioned earlier, Ag-NPs and more specifically Ag^+^, react with thiol groups [5, 38, 47]. Thiol is the functional group on the amino acid cysteine. Cysteine is very important in biological reactions due to disulfide bridging which is crucial for proper protein folding and function, as well as, its nucleophilic role in catalytic reactions. When adding cysteine to a mixture of Ag^+^ and bacteria, the antibacterial activity of Ag^+^ is neutralized, indicating an interaction of Ag^+^ with thiol groups [47, 85]. It is important to highlight that there are thiol groups in essential pathways such as respiratory and cell wall synthesis enzymes, which represent potential locations of Ag^+^ binding [88]. In the specific case of cell wall synthesis enzymes, it has been reported that the protein-NP interaction occurs in the SH group of the mannose phosphate isomerase, leading to an interruption of cell wall synthesis with a concomitant leaching of internal components, and cell death [5].

The hypothesis that Ag^+^ binds to the DNA was confirmed after the observation that bacterial DNA was condensed when both *E. coli* and *S. aureus* species were exposed to Ag^+^, leading to a consequent cell multiplication arrest [3]. High-resolution imaging revealed a low-molecular-weight region (low density region) formed in the center of the bacteria, suggesting that this is a mechanism of defense employed by the bacterial cell as a result of Ag^+^ exposure. This phenomenon suggests that the bacterium senses either a disturbance in the cell membrane or the presence of a threat such as Ag^+^ and condenses its DNA to protect it from potential incoming damage [3]. Surprisingly, when Ag-NPs were used in place of Ag^+^ in *E. coli* cells, the condensation did not occur [40]. This suggests that the bacterial cell may sense the presence of a threshold of Ag^+^ to activate the mentioned defense mechanism. On the other hand, as a result of the contact of Ag-NPs with the cell, the Ag^+^ concentration is insignificant or below the required concentration to activate the defense system.

Many studies exposing cells to NPs found that the DNA was damaged [29, 71, 86]. This damage included nuclear fragmentation [72] or physical attachment of the Ag-NPs to the DNA, probably because of the high affinity of Ag^+^ to phosphates highly abundant in the DNA molecule [40].

## Global gene and protein regulation upon exposure to NPs

NPs exposed to bacterial cells have been shown to cause changes in the genomic and proteomic profiles, suggesting that the presence of NPs primes an adaptation of the cells to the new NP-containing environment. For example, when Ag-NPs and Ag^+^ were exposed to bacterial cells, an upregulation of a shared 161 genes and downregulation of 27 genes in *E. coli* were observed. Interestingly, Ag-NPs and Ag^+^ exclusively regulated 309 and 70 genes, respectively [70]. Another study reported that *E. coli* treated with Ag-NPs upregulated many genes covering a wide range of functions such as membrane structure and biofilm formation (*bolA*), the citric acid cycle (*sdhC*), electron transfer (*sdhC*), cellular transport (*mdfA*), protein efflux (*fsr*, *yajR*, *emrE*), and DNA repair (*recN*, *uvrA*, *ybfE*, *yebG*, *ssb*, *sbmc*, and *nfo*) [87].

In the case of CeO_2_-NP exposure to *E. coli*, 144 genes were differentially expressed [16], particularly with a higher expression of *cydA* and *cydB* transcripts, which encode for the cytochrome terminal oxidase subunits I and II. Most other changes in expression levels seemed to indicate that Ce disrupts respiration or iron homeostasis because many iron uptake genes responded to NP treatment [16].

*Escherichia coli* treated with MgO-NPs differentially regulated 109 proteins with 83 being downregulated [68]. These proteins were mostly part of central metabolism, genetic transcription, and others needed for cellular function. The upregulated genes were thiamine-binding periplasmic protein and proteins associated with riboflavin metabolism, suggesting that the upregulated genes did not seem to bear relevance to the toxicity of MgO-NP exposure.

### ROS and metabolism gene regulation

The increasing ROS level in a bacterial cell will induce the transcription of genes involved in the cellular protection against ROS. In contrast, not all the NPs are able to elicit an antioxidant response as in the case of *E. coli* exposed to MgO-NP [68].

*Pseudomonas* sp. cells treated with Ag-NPs upregulated the expression of the following proteins: translational ribosomal proteins S2 and L9, ketohydroxyglutarate aldolase (KHGA), AhpC (alkyl hydroperoxide reductase) and TSA (thiol-specific antioxidant) [88]. Both TSA and AhpC belong to an antioxidant family of enzymes called peroxiredoxins, which protect the cell from peroxide damage and are expressed during an oxidative stress [89]. This upregulation supports the hypothesis that Ag-NPs induce oxidative stress in cells because of the increasing level of these enzymes produced to cope with the increasing ROS levels. KHGA is associated with sugar metabolism, converting sugar acids, hexonates, and hexuronates into pyruvate and glyceraldehyde-3-phosphate [90]. It also regulates glyoxylate levels and prevents toxin accumulation [91]. KHGA may be expressed due to the Ag-NP induction of metabolic change. Translational ribosomal proteins S2 and L9 are involved in translational regulation and also have functions in structure and stress regulation [92].

In the case of *E. coli* exposed to TiO_2_-NPs, an upregulation of the enzyme *aphF* was observed [87]. Both *ahpC* (upregulated by Ag-NPs) and *aphF* are involved in peroxide metabolism, but are differentially regulated upon exposure to different NPs. This suggests that different pathways for upregulation are involved [88]. A similar observation of *ahpC* downregulation was reported in another study when *E. coli* was exposed to TiO_2_-NPs [34].

Another gene triggered by high peroxide levels is *katE*, a catalase that decompose H_2_O_2_ to protect the cell from ROS damage. When the gene *katE* is absent (gene knock out), an Ag-sensitive phenotype is induced [30]. *oxyR* is another gene upregulated upon oxidative damage response by exposure to Ag-NP. This gene regulates redox reactions and is involved in peroxide metabolism and protection [87]. Other genes involved in these processes that were found to be upregulated are *sodA*, *sodB*, *sodC*, and *katG*. All of these genes work together in an oxidative species reaction, turning ‎O^2−^ to H_2_O_2_ and then into the harmless O_2_. After 90 min exposure, *oxyR* production began to decline [87]. This could be due to a feedback loop of the protein regulating the gene or it could be due to progressive cell membrane disintegration, leading to the entire cell no longer being able to regulate gene expression.

### ATP inhibition

The production/recycling of ATP in bacterial cells exposed to ROS as a result of NP activity is compromised. For instance, *E. coli* cells treated with Ag^+^ inactivate the expression of ribosomal subunit proteins as well as other cellular proteins and enzymes essential to ATP production [93]. Most notably, the expression of S2 protein that is a subunit of the 30S ribosome is decreased by Ag^+^, which causes the ribosome to lose its function and essentially denature [93].

The 30S subunit is responsible for proper base pairing between the codons and anticodons. As a result of its denaturation, the expression of other proteins are suppressed, such as succinyl-CoA synthetase which is necessary for catalysis of intracellular ATP production [93]. The deficiency in necessary proteins and enzymes to run the citric acid cycle leads to a deficiency in ATP. This could explain the ATP depletion observed upon exposing *E. coli* to Ag-NPs, and is supported by the fact that ATP content in the *E. coli* cells was depleted even though there was no ATP detected in the media, meaning that depletion was not due to leakage [55].

### Stress condition proteins

Bacteria are exposed to stress originating by multiple sources in the environment. To adapt and survive the stress, bacteria respond by activating and coordinating a complex network of genes that cope with the external stimulus for an effective response. Two of the most important stress responses include the upregulation of envelope stress and heat shock proteins. Both have been observed when bacterial cells have been treated with NPs.

It has been found that the expression of cell envelope proteins seems to be upregulated upon Ag-NP exposure. This was detectable because the proteins remained in a precursor form due to the Ag-NP inhibition of the process of conversion into shorter, mature forms in *E. coli* [55]. The conversion requires a membrane potential and ATP, especially for cleavage and translocation of the mature proteins into the periplasm and outer membrane. However, both of these requirements were abolished upon Ag-NP treatment. Examples of envelope proteins that began to accumulate in the cytoplasm include the outer membrane proteins OmpA, OmpC and OmpF, periplasmic oligopeptide binding protein A (OppA), and d-methionine binding lipoprotein (MetQ) [55]. OmpF was also upregulated when *E. coli* was exposed to TiO_2_-NPs [34]. Other studies reported controversial reports such as a global downregulation of Omp proteins [47, 70].

Cell envelope gene regulation requires more elucidation, as most results seem contradictory. In addition to differences between reported Omp protein activities, it is unclear how the membrane protein regulation reacts to NP exposure. In this regard, the regulation of genes involved in the synthesis of other biomolecules such as lipids and fatty acids are downregulated despite a stimulation occurring as a result of membrane damage [70]. Even though the membrane is being ruptured, membrane proteins and transport-associated proteins Cmr, Fsr, YajR, and EmrE are upregulated upon Ag-NP exposure [87]. To further contradict this finding, it has also been reported that in the absence of inner membrane proteins DcuC, SdhD, TatC, TolR, TonB, and TrkA [30], an Ag sensitive phenotype was established, despite the findings of cellular membrane upregulation [87].

Heat shock proteins combat stress and are induced when protein denaturation is sensed through their chaperone functions [69, 92, 94]. For example, the heat shock genes encoding inclusion body binding proteins A and B (*ibpA* and *ibpB*), *groL* and *groS*, and the 30S ribosomal subunit S6 are upregulated upon Ag-NP exposure [70]. Other genes encoding for chaperonins were upregulated as in the case of *dnaK*, *dnaJ*, and *grpE* [70]. With so many heat shock response genes being regulated, it is likely that Ag^+^ acts on protein structure priming the stress response mechanism.

### Effect of NPs on sulfur-related proteins

An upregulation of genes involved in sulfur metabolism has been observed upon exposure of bacterial cells to NPs, suggesting that perhaps there is linkage between sulfur and NPs. CeO_2_-NPs tested against *E. coli*, *B. subtilis*, and *S. oneidensis* yielded findings that genes *rnt*, *thiS*, *cysI*, *cysN*, *cysW*, *yciW*, *ilvG*, and *pyrB* were differentially expressed between NP exposure and salt exposure (osmotic stress) [16]. The majority of these genes are related to sulfur metabolism, including the subunits of ABC family sulphate/thiosulphate transporter as well as genes required for intracellular sulfate reduction and assimilation during cysteine synthesis. All of these genes are induced upon Ag^+^ exposure. One of the main reasons for this upregulation may be the increased demand of cysteine as this residue is a target for Ag^+^ and its intracellular depletion results in an activation of its biosynthetic pathway [70].

Iron–sulfur (Fe–S) proteins contain Fe–S clusters, which are found in a variety of proteins, such as metalloproteins, hydrogenases, bacterial respiratory complexes I–III, succinate-coenzyme Q reductase, and ferredoxins [95]. It has been reported that Ag^+^ induced operons *isc* and *suf*, the genes responsible for encoding Fe–S clusters [70], whereas controversially the genes *iscX* and *hscB*, both associated with the formation of Fe–S clusters, were found to be downregulated significantly when *E. coli* cells were exposed to TiO_2_ [34].

### Toxicity of Cu-NPs

Ag^+^ is isoelectronic to Cu^+^ with the two cations having the same ionic radii, charge, and d10 electronic configuration [20]. However, since Ag^+^ is a non-essential metal and Cu is an essential microelement, the exposure of Ag-NPs has been found to trigger Cu related gene regulation [47, 69]. Ag^+^ can potentially interact with Cu sensor proteins *cusS* and *cueR* which in turn activate Cu^+^ regulation genes *cusCFBA*, *copA* and *cueO* [70]. *CusR* upregulates the *cusCFBA* operon which encodes an anti-porter efflux transporter; this upregulation increases with Ag-NP exposure when compared to Ag^+^ exposure regardless of ion concentration [48]. *CusS* helps regulate *cueR* synthesis, which triggers a Cu resistance mechanism, but the observed upregulation upon Ag-NP and Ag^+^ exposure supports the hypothesis that Ag^+^ and not Cu alters *cusS* and *cueR* expression [30].

The genes *copA*, *cueO*, and *cusA* are upregulated upon exposure to Ag-NPs in *E. coli* [48]. These genes are associated with Cu^+^ homeostasis and stress, but have been linked to Ag^+^ stress response as well. *CopA* upregulation is indicative of a high Ag^+^ level in the cytoplasm [48]. Interestingly, the protein profile is similar to that of exposure to Cu-NPs; these genes associate with ATPase activity and periplasmic concentrations and it is possible that the cells do not discriminate between both cations, leading to the same ROS management response with Ag^+^ as in Cu^+^ [48].

### Effect of NP on DNA replication and repair

*Escherichia coli* exposed to TiO_2_-NPs downregulated genes *dnaX* and *holB*, both involved in DNA replication [34]. Downregulation of genes involved in induction of purines (*guaC*), pyrimidines (*pyrC*), and glutaredoxin, an amino acid cofactor (*grxA*), indicates the downregulation of DNA synthesis as a response to TiO_2_-NP exposure. This suggests that the cell is under stress and not prioritizing DNA synthesis [34]. Many genes associated with amino acid transport (*argT*, *glnH*, *livK*, *tdtC*) and glutamine synthesis (*glnA*) are also upregulated and potentially reflect a cell attempting to respond to an environmental adaptation [34].

TiO_2_-NPs were also tested and various DNA repair genes were stimulated, including: *recN*, *mutT*, *nfo*, *uvrA*, *uvrD*, *umuD*, *polB*, and *ssb*. This means that the DNA is damaged upon exposure to metal NPs, but different mechanisms are triggered to respond to the damage.

Interestingly, the gene *recA* is expressed during DNA damage and presents as an Ag^+^ treated phenotype when downregulated [30]. It is unclear whether Ag^+^ directly downregulates the gene to prevent DNA repair or if it is a result of other toxicity mechanisms. For example, *E. coli* cells treated with Ag-NPs did not suffer any global protein change, however, specific protein groups showed a change in regulation. The Ag-NPs have selectivity when binding to protein groups, but do not bind enough to alter protein–protein interactions on a global scale in the cells [55].

## Conclusions

**Fig. 4:**
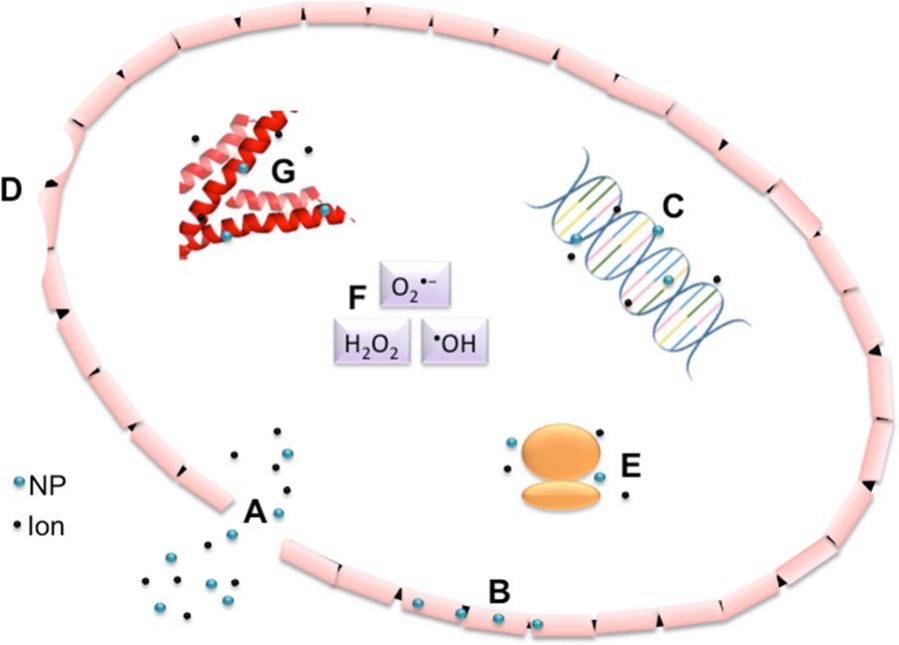
A proposed model showing the mechanisms of action of Ag-NPs exposed to Gram-negative *E. coli* cell. (A) Disintegration of cell wall allowing intracellular components to leave the cell. (B) Ag-NPs entering periplasmic space, beginning a separation of the cytosol from membrane. (C) Interaction of Ag-NPs with DNA. Inhibition can cause ROS production. (D) Cell pits occurring after exposure. (E) Inhibition of proper ribosome function, leading to ROS production, malformation or suppression of proteins, improper DNA function. (F) ROS production. (G) Interaction with proteins, specifically cysteine

It is evident in the literature that both NPs and specific ions exhibit strong antibacterial activity. The exact mechanism through which this activity occurs is only hypothesized and needs to be studied further. Although the multiple pathways that seem to be simultaneously activated by NPs make elucidation a difficult task, they are also the reason why NP exposure is so effective. The combination of ROS production, gene regulation changes, cell wall penetration, and metabolite binding are challenges for adaptation and survival, and the bacteria fail to establish a defense simultaneously against all of the interactions (Fig. 4). After reviewing the literature, it seems that it is indeed this combination itself that causes the toxicity, and not likely that one single factor is responsible for the bacterial killing. Although these mechanisms would also be toxic to human cells because of the similarity of the biomolecules (lipids, proteins and DNA), potential treatments of bacterial infections could be targeted focally by using specific ligands and bacterial cell receptors. The multi-target activity caused by NPs would be ideal to treat and kill multi-drug resistant bacteria, as they likely would not be able to mount multiple defenses at once. Before future application can be explored, more research should be done to gain a further understanding of how the antibacterial system functions upon exposure to NPs, with elucidation of hypothesized activity and investigation into new potential mechanisms.

### Future venues

As antibiotics continue to be misused, overprescribed, and used extensively in husbandry practices, the state of antibiotic resistance will only worsen. Lack of novel treatments contributes to the worsening situation, as bacteria with developed resistance are able to replicate freely with no effective management. Although NPs are a potential solution to this issue due to their multi-target mechanism of action, more work must be done. Before regular NP medical application occurs, a standardization of formulation, characterization, and testing must be put in place. Due to the variation of NP protocols in the literature, it is hard to corroborate the current existing studies to a result that may progress to an antibiotic alternative. In addition, few studies examine NP effect on human cells. It is important for cytotoxicity and immune response to be investigated alongside medical application to find a balance between the concentration necessary for desired activity and minimized cytotoxicity and immune response. NP levels have been found to be toxic around 5–10 μg/mL in eukaryotic cells. If effective antimicrobial concentrations are higher than cytotoxic levels, this could be problematic for practical use.

In conclusion, standardized practices in NP fabrication should be considered for maximal validation amongst future studies, which should include a cytotoxicity analysis and an inflammatory response. Moreover, the emerging number of multiple-drug resistant bacterial strains should be addressed by testing clinical isolates rather than traditional strains from microbial collections.

## Abbreviations

AhpC: alkyl hydroperoxide reductase
BPEI: branched polyethylene imine
CTAB: cetyl trimethylammonium bromide
GSH: reduced glutathione
GSSG: oxidized glutathione
KHGA: ketohydroxyglutarate aldolase
MetQ: d-methionine binding lipoprotein
NP: nanoparticle
Omp: outer membrane proteins
OppA: periplasmic oligopeptide binding protein A
PVA: polyvinyl acetate
PVP: polyvinyl pyrrolidone
ROS: reactive oxygen species
TSA: thiol-specific antioxidant
